# Examining Host Selection by Mexican Bean Beetle (Coleoptera: Coccinellidae) Using Mark-Release-Recapture

**DOI:** 10.1093/jisesa/iez007

**Published:** 2019-02-17

**Authors:** Louis B Nottingham, Thomas P Kuhar

**Affiliations:** Department of Entomology, Virginia Tech, Blacksburg, VA

**Keywords:** *Epilachna*, *Phaseolus*, host, preference, cultural, cultivar

## Abstract

Mexican bean beetle, *Epilachna varivestis* Mulsant, is a pest of snap bean and lima bean in the eastern United States. This pest is susceptible to many insecticides available to conventional growers; however, organic management using parasitoid releases and organic insecticides have inconsistent results. In the interest of developing cultural management techniques such as trap crops or push–pull systems, five bean cultivars were evaluated for preferential host selection by *E. varivestis* using marked beetles in field cages and open plots. Beetles were marked with a water-based paint pen and their locations on plants monitored over time. In field cages, the purple wax bean, Dragon’s Tongue (DT), was preferred over yellow wax, green bean and lima bean; soybean was the least preferred overall. Recaptures of *E. varivestis* adults in open field plots progressively decreased following beetle release, suggesting the affinity of adults to disperse despite being on or near acceptable hosts. The two wax beans were equally preferred in open field experiments, partially more than the green and lima bean and consistently more than the soybean. These experiments suggest that DT may be a suitable crop for trap cropping or attract and kill strategies for *E. varivestis*.

Mexican bean beetle, *Epilachna varivestis* Mulsant (Coleoptera: Coccinellidae), is an herbivorous ladybeetle that feeds on many wild and cultivated legumes (Fabaceae). This native of western Mexico and Central America (Chittenden 1919, Howard and English 1924) has been a pest of snap bean, *Phaseolus vulgaris* L., (Fabales: Fabaceae), lima bean, *Phaseolus lunatus* L., (Fabales: Fabaceae), and soybean, *Glycine max* (L.), (Fabales: Fabaceae), in the eastern United States for nearly a century (Nottingham et al. 2016). The pest is most common and severe in the southern Appalachian Mountains and Mid-Atlantic regions of the United States, particularly on organic farms where insecticides are not often used (Nottingham et al. 2016).

Overwintered adults enter bean fields from surrounding woodlands in late spring to feed and lay eggs (Howard and English 1924). Adults and larvae feed on the leaves of young plants, leading to yield reductions after ~20% defoliation (Capinera et al. 1987, Capinera 2001). Once pods form, beetles feed on the outer tissue creating scars and disfigured fruit (Capinera 2001, Nottingham et al. 2016). Beetles complete two to four generations in a season depending on the region. Newly emerged adults of each generation are inclined to disperse and have been documented flying ~20 ha to find new bean hosts or overwintering sites (Auclair 1960).

*Epilachna varivestis* can be effectively controlled with synthetic insecticides such as pyrethroids and neonicotinoids (Shamiyeh and Mullins 1987, Nault and Speese 2001, Patton et al. 2003, Nottingham and Kuhar 2017). Augmentative release of the parasitoid, *Pediobius foveolatus* (Crawford) (Hymenoptera: Eulophidae), has also shown success in reducing *E. varivestis* pest densities (Fess 2008, Nottingham et al. 2016). However, pest control tactics (especially selective methods) are more likely to succeed if implemented in addition to a prophylactic, cultural strategies (Gray et al. 2009).

Cultural tactics often involve strategic planting schemes that disrupt a pest’s potential to injure a crop (Kogan 1988). Snap bean and lima bean cultivars are known to have variable susceptibility to *E. varivestis* (Howard and English 1924, Friend and Turner 1931, Campbell and Brett 1966, Raina et al. 1978, Flanders 1984), which could provide a platform for cultural techniques. Although planting resistant host plants is one strategy for cultural management, dynamic arrangements of resistant and susceptible plants, such as trap crops (attractive crop zones grown to consolidate pests in a location) and push–pull (using both attractants and repellents to consolidate pests in a location), can increase the control potential while adding crop diversity (Hokkanen 1991, Gray et al. 2009). Rust (1977) tested a trap cropping strategy for *E. varivestis* using the snap beans (cv. ‘Blue Lake’) as a trap for lima beans and soybean (cultivars chosen by growers and not documented). While soybeans incurred low injury, lima beans were not protected. To our knowledge, that is the only published study testing trap cropping for *E. varivestis*. Most snap bean cultivars are preferred over lima beans cultivars by *E. varivestis*, but occasionally the opposite occurs (Campbell and Brett 1966, Raina et al. 1978). Therefore, it is necessary to evaluate and select candidates for trap cropping by cultivar, not species.

The study presented herein measured preferential host selection of *E. varivestis* among five bean cultivars (three snap, one lima, and one soybean). Our goal was to use this information to determine the potential for these cultivars in trap cropping or push–pull strategies. The cultivars tested (‘Dragon’s Tongue’ [DT] purple wax snap, ‘Rocdor’ yellow wax snap, ‘Caprice’ green snap, ‘Fordhook’ bush lima, and ‘Hutchison’ field soybean) were chosen based on our corresponding study measuring cultivar susceptibility, in which we determined that each of the cultivars exhibit significantly different levels of attraction for oviposition by wild *E. varivestis* in field plots (Nottingham and Kuhar, in press). Here we examine two specific factors of host attraction important to trap cropping and push–pull strategies: 1) the ability of a cultivar to lure beetles out of another acceptable host cultivar; and 2) the ability of a cultivar to retain beetles from moving into other cultivars or offsite. To examine these factors, marked beetles were evenly distributed into field plots of each cultivar, and their movements were tracked over the course of 5 d. We hypothesized that *E. varivestis* would distribute based on preferences, resulting in aggregations in the preferred cultivar(s) and deficits in less preferred cultivars. Cultivars with significant differences in attraction are likely candidates for trap cropping and push–pull designs.

## Materials and Methods

### Plots

**Fig. 1.**
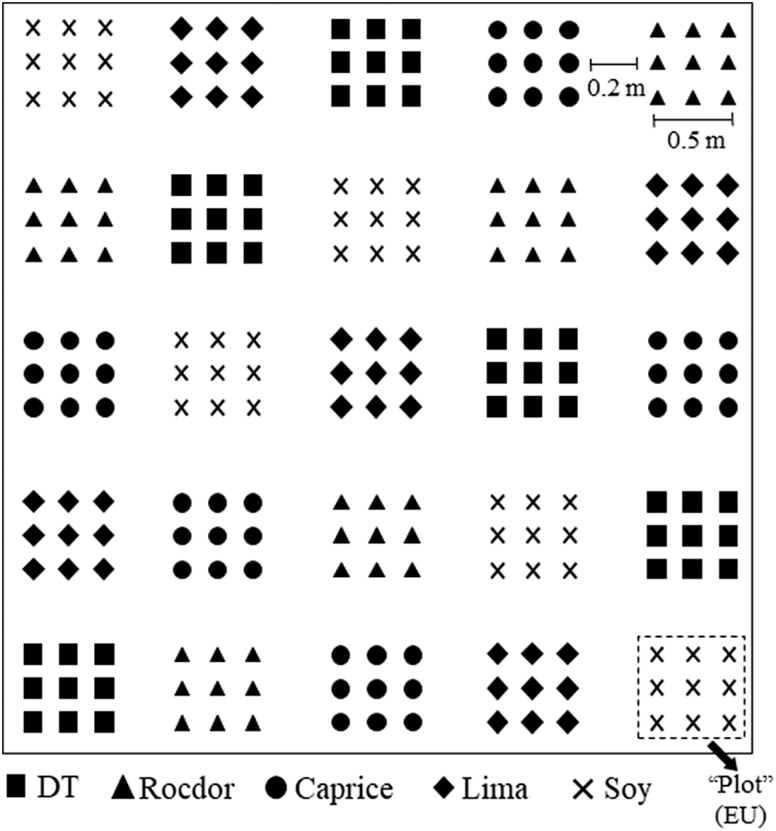
Plot design of mark-release-recapture arenas for measuring *E. varivestis* cultivar choice. Two identical arenas were established in both years, the only difference being that one was covered in mesh cage; the other was not. Individual symbols represent a single plant. Nine plants of the same cultivar in a group are denoted as a ‘plot’, or experimental unit (EU). Five marked beetles were released into each plot at the beginning of each experiment (125 beetles/arena/experiment).

Mark-release-recapture experiments were conducted at Virginia Tech’s Kentland Farm in Whitethorne, VA. Four separate experiments were conducted, two each in 2013 and 2014. Each year, one experiment was run in an open field arena and the other under a field cage of polyethylene mesh draped over a steel frame (3.7 × 3.7 × 2.5 m). Cages were used to prevent beetles from escaping the experimental arena; but because the cage would potentially affect behavior, it was also necessary to have an uncaged arena for comparison. The cage was set up 1 d prior to the release of beetles to avoid major differences in plant growth among arenas. Arenas consisted of five bean cultivars grown from seed, in the arrangement of 25 experimental units (plots). Plots were arranged in a randomized complete block, with five replicates per cultivar randomized by column (Fig. 1). The cultivars tested were Caprice (bush green snap bean, *P. vulgaris* L.), Rocdor (bush yellow wax snap bean.), DT (bush purple wax snap bean), Fordhook (bush lima bean, *P. lunatus* L.), and Hutcheson (field soybean, *G. max*). The arrangement of cultivars was identical in cage and no-cage arenas within each year. Beans were sown on 14 August 2013 and 2 August 2014. Arenas were at opposite ends of a 120-m-long planting bed. Intermediate row space separating arenas was a mixed grass cover crop. The closest other planting of beans was greater than 300 m from either arena.

#### Mark-Release-Recapture Methods

Mexican bean beetle pupae were collected in the field from untreated snap bean plants (cv. ‘Provider’), by clipping whole leaves with numerous pupae. Pupae were put into mesh cages (still on leaves) (30 × 30 cm, 1466AV, BioQuip Products, Inc., Rancho Dominguez, CA) containing potted scarlet runner beans (*Phaseolus coccineus* L.). Once adults emerged, they were left to feed on runner beans for 1 wk until elytra hardened. Beetles were sorted by sex using the presence or absence of a notch at the end of the eighth abdominal sternite (Sweetman 1932), and males were discarded. Five beetles at a time were put in a zipper-locking bag and placed in a freezer (−18°C [0°F]) for about 1 min to aid handling and marking. Each adult female was marked with one of five colors on the right elytron, using a water-based paint pen (Sharpie USA, Downers Grove, IL). Each color represented the bean cultivar in which the beetle would be released. After being marked, beetles were placed back into cages with runner beans to feed for another 5 d, so that any beetles injured or killed by the procedure could be discarded.

Prior to formal experiments, a preliminary trial was performed to examine if marking procedures would kill beetles or disrupt their dispersal behavior. The same marking procedure described above was performed. One hundred fifty beetles were marked (30 per color) and kept in cages with runner beans. Cages were checked daily for 5 d after marking for living and dead beetles. Twenty-three beetles died overall, most (20) in the first 24 h. Therefore, it was concluded that beetles surviving past 24 h were healthy enough for experimentation (death was likely the result of poor handling). Fifteen beetles of each color (75 total) were taken to the field and released into mixed grass/weedy vegetation ~15 m away from a plot of untreated snap beans. After 24 h, about 30% of the beetles were recovered in the nearby snap beans, and only one was found (dead) at the release site. Of the beetles recaptured, all marking colors were represented without any noticeable bias. These tests led us to conclude that marking procedures would not have a major impact on beetle dispersal behavior.

Beetle releases for formal experiments occurred on 12 September 2013 and 27 August 2014. Immediately prior to releases, all plants in arenas were searched for wild *E. varivestis*, which were removed. On the day of release, beetles were separated by color and taken to the field. Five beetles were released into each plot (25 beetles/cultivar/arena, 125/arena, 250/year). Releases were made by carefully placing beetles in the canopy of the center plant for each plot. Beetles were observed for at least 1 min after release, returning or replacing any beetles that dropped or flew away.

Plots were visually searched for marked beetles at 24, 72, 120, and 168 h after release. Only marked beetles were counted; beetles without marks were removed from arenas. Recaptured beetles were not handled, and left at the spot of recapture. If beetles dropped to the ground, they were returned to the leaf on which they were found using a plastic spoon.

Recaptured beetles were categorized as either *immigrated* (from a different cultivar) or *retained* (from the same cultivar), to be used as dependent variables for analysis. The sum of immigrated and retained recaptures (*combined*) was the third dependent variable. Separating recaptured beetles into these subcategories provided more specific metrics of beetle-attraction to each cultivar: 1) ability to attract dispersing beetles (immigrated), and 2) ability to prevent dispersal into other hosts (retained). Combined recaptures provide the overall outcome of both types of selection.

### Data Analyses

All data were analyzed using SAS (SAS Institute Inc. 2018). Three dependent variables, immigrated, retained and combined (sum of immigrated in retained) recaptured beetles/plot, were analyzed separately for each test. Cage and no-cage data were analyzed separately due considerable differences in recaptures between the two arena types (no-cage arenas having 67% lower recovery). Due to very few recaptures of immigrated beetles in the 2014 no-cage arena, those data were omitted from the no-cage immigrated model (i.e., for no-cage arenas, immigrated recaptures only comprised 2013 data, while retained and total recaptures comprised 2013 and 2014 data). Initially, main effects were tested using repeated measures (proc glimmix) with cultivar and day as fixed effects. Despite several manipulations of the model (blocking factors and covariance structures), G-matrix was not positive and definite. Therefore, mixed model analysis of variance (ANOVAs; proc mixed) were used to determine significant effects of cultivar (days pooled) and day (cultivars pooled) separately, using rep as the random (blocking) factor for both. Heterogeneity of variance and non-normality of errors were evaluated by comparing residual/predicted plots (confirm lack of cone-shaped pattern) and fit statistics (approving lower AICc values) among raw data and nonlinear transformations. Raw data resembled a linear distribution, and therefore, transformations were unnecessary. Significance of main effects were accepted at *P* < 0.05. Significant differences (*P* < 0.05) among means were determined using Tukey’s HSD multiple comparisons test (adjust=tukey option of the lsmeans statement).

## Results and Discussion

**Fig. 2.**
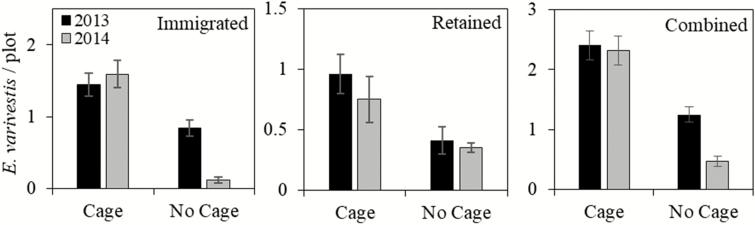
Average number of recaptured *E. varivestis* per plot (±SEM) for cage and no-cage arenas by year (2013 and 2014) and for each recapture type (immigrated, retained and combined recaptures). Recapture averages were pooled across cultivars and sample days. Cage and year were not compared statistically because only two replicates were used for each.

**Fig. 3.**
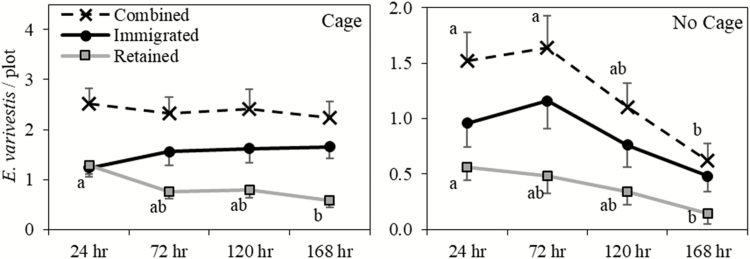
*E. varivestis* recaptures by sample day (hours after release) for cage and no-cage arenas. Data were pooled across cultivars. Each marker indicates average *E. varivestis* recaptures per plot (±SEM) for each sample by recapture type (immigrated, retained, and combined recaptures). Markers on the same line and in the same chart not sharing a letter are significantly different (*P* < 0.05) according to a Tukey’s HSD multiple comparisons test.

The caged arenas resulted in greater beetle recaptures overall (all other factors pooled) compared with uncaged arenas by 48% in 2013 and 80% in 2014 (Fig. 2). In caged arenas, both recapture types (immigrated and retained) were similar from year to year (Fig. 2). In no-cage arenas, immigrated recaptures decreased by 85% in 2014, while retained recaptures remained similar (Fig. 2). Recaptures by sample date (years and cultivars pooled) showed that combined recaptures decreased significantly in no-cage arenas, but were stable in caged arenas (Table 1, Fig. 3). When broken into retained and immigrated recaptures, retained recaptures decreased significantly over time for both caged and no-cage arenas (Table 1, Fig. 3). Immigrated recaptures decreased noticeably, although not significantly, in the no-cage arena, and were stable in the caged arena (Table 1, Fig. 3).

**Table 1.** Effects of independent variables, sample day and bean cultivar, on numbers of *E. varivestis* recaptures (immigrated, retained and combined recaptures) per experimental unit (plot), for both cage and no-cage arenas

	Immigrated			Retained			Combined Recaptures		
Variable	df	*F*	*P*	df	*F*	*P*	df	*F*	*P*
Cage									
Day	3,12	1.46	0.275	3,12	5.28	0.015	3,12	0.29	0.835
Cultivar	4,16	54.65	<0.001	4,16	12.78	<0.001	4,16	37.04	<0.001
No-cage									
Day	3,12	1.98	0.171	3,12	8.95	0.002	3,12	6.0	0.010
Cultivar	4,16	12.35	<0.001	4,16	5.52	0.006	4,16	12.80	<0.001

Prior to analysis, data were pooled across 2013 and 2014. Immigrated recaptures in the no-cage arena only used 2013 data due to low overall recapture in 2014. Mixed model ANOVAs with replicate as the random variable were performed for day and cultivar independently. Data were pooled across cultivars to test the effect of day, and across days to test the effect of cultivar. *P* values < 0.05 indicate a significant effect.

The decreasing trend in no-cage arena recaptures from 2013 to 2014 may suggest that beetles were more prone to dispersal following release in 2014. Caged arena immigrated recaptures did not change from 2013 to 2014; therefore, the cage either prevented the causal agent of dispersal behavior (sunlight, wind, etc.) or blocked beetles’ dispersal path. The latter theory is supported by the increase in immigrated recaptures and decrease in retained recaptures in 2014s caged arena (Fig. 2). Beetles trying to disperse from the arena were blocked by the cage, forcing them back into the arena where a cultivar choice was made. Thus resulting in greater immigrated recaptures and fewer retained recaptures.

**Fig. 4.**
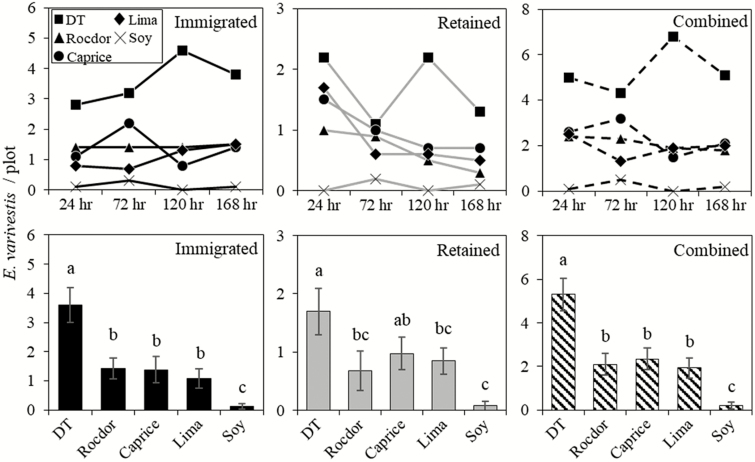
Top graphs show caged arena recaptures of *E. varivestis* in each cultivar by day. Daily comparisons were not analyzed statistically. Bottom graphs show caged arena average *E. varivestis* recaptures per plot (±SEM) by cultivar, pooled across samples days. Cultivar averages not sharing a letter are significantly different (*P* < 0.05) according to a Tukey’s HSD multiple comparisons test.

**Fig. 5.**
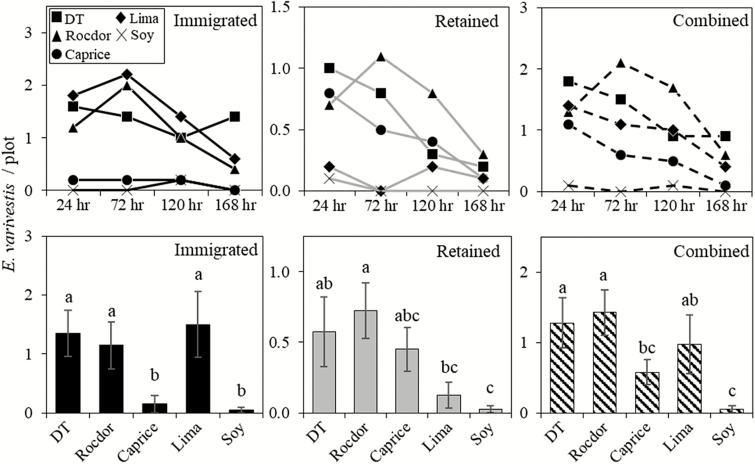
Top graphs show no-cage arena recaptures of *E. varivestis* in each cultivar by day. Daily comparisons were not analyzed statistically. Bottom graphs show no-cage arena average *E. varivestis* recaptures per plot (±SEM) by cultivar, pooled across samples days. Cultivar averages not sharing a letter are significantly different (*P* < 0.05) according to a Tukey’s HSD multiple comparisons test.

Recaptures in each cultivar are shown by date (Figs. 4 and 5, upper graphs) and pooled across dates (Figs. 4 and 5, lower graphs). Immigrated, retained and combined recaptures were significantly different among cultivars for both caged and no-cage arenas (Table 1). For the caged arena, the differences in recaptured beetles among cultivars were the same for immigrated and combined recaptures. DT had significantly more immigrated and combined recaptures than all other cultivars; Caprice, Rocdor, and lima had similar for each recapture type; soybean had the fewest of each recapture types (Fig. 4, bottom left and right). Retained recaptures exhibited a similar trend, but with intermediates (Fig. 4, bottom center). The no-cage arena had lower recaptures overall, greater variability, and was therefore less clear in terms of cultivar preference. Overall, the wax beans, DT and Rocdor, were most preferred, having high-retained and immigrated recaptures (Fig. 5). Lima and Caprice had mixed outcomes for immigrated and retained recaptures, and soy had consistently low recaptures of both types (Fig. 5).

In conclusion, discriminatory host preference by *E. varivestis* was observed in both cage and no-cage arenas. Caged arenas had higher recovery rates due to their ability to prevent beetles from escaping, thus providing clearer choice results than no-cage arenas. Combined recaptures alone suggest that DT was the most preferred cultivar overall, having approximately 55% more recaptures than the next closest cultivar, Rocdor. Soybean was clearly the least preferred, with the lowest recaptures for all trials and factors. These results reflect past studies demonstrating the preference of *E. varivestis* to wax beans over most green snap bean and lima beans cultivars, and all soybeans (Friend and Turner 1931, Campbell and Brett 1966, Raina et al. 1978, Nottingham and Kuhar in press). DT had more immigrated recaptures than all other cultivars, suggesting that it was the most attractive cultivar to dispersing *E. varivestis*. DT also had the most retained recaptures overall, suggesting that beetles in DT were the least likely to disperse. Our experimental design did not allow us to determine whether retained beetles actually remained in their original plot, or just the cultivar as a whole. Retained recaptures may have been the result of beetles exiting and re-entering their initial plot or moving to a different plot of the same cultivar. Either of these situations could explain why DT plots in the caged arena had a ~50% drop in retained recaptures at the 72-h sample, followed by an equivalent rebound at 120 h (Fig. 4, top center). Although DT was the most preferred of the cultivars evaluated, enough beetles were found in all other *Phaseolus* cultivars to suggest that more experimentation is necessary to successfully implement a trap crop strategy for *E. varivestis* using DT and one of the less preferred cultivars. Advancing this strategy to a dead-end trap crop, push–pull system or both would likely improve its potential for control. For example, planting Caprice on reflective plastic mulch which repels beetles (Nottingham and Kuhar 2016) adjacent to DT treated with an insecticide.
